# "To Bluff like a Man or Fold like a Girl?" – Gender Biased Deceptive Behavior in Online Poker

**DOI:** 10.1371/journal.pone.0157838

**Published:** 2016-07-06

**Authors:** Jussi Palomäki, Jeff Yan, David Modic, Michael Laakasuo

**Affiliations:** 1 Cognitive Science, Institute of Behavioural Sciences, University of Helsinki, Helsinki, Finland; 2 School of Computing & Communications, Lancaster University, City of Lancaster, United Kingdom; 3 Computer Laboratory, University of Cambridge, Cambridge, United Kingdom; University of Tuebingen Medical School, GERMANY

## Abstract

Evolutionary psychology suggests that men are more likely than women to deceive to bolster their status and influence. Also gender *perception* influences deceptive behavior, which is linked to pervasive gender stereotypes: women are typically viewed as weaker and more gullible than men. We assessed *bluffing* in an online experiment (N = 502), where participants made decisions to bluff or not in simulated poker tasks against opponents represented by avatars. Participants bluffed on average 6% more frequently at poker tables with female-only avatars than at tables with male-only or gender mixed avatars—a highly significant effect in games involving repeated decisions. Nonetheless, participants did not believe the avatar genders affected their decisions. Males bluffed 13% more frequently than females. Unlike most economic games employed exclusively in research contexts, online poker is played for money by tens of millions of people worldwide. Thus, gender effects in bluffing have significant monetary consequences for poker players.

## Introduction

Deception and dishonest signaling—implicit or explicit acts to propagate false information—can be observed throughout the animal kingdom [1]. In humans, deception is a part of the “flip side” of cooperation in communal living [2]: through evolution, we learned to cooperate, but also to manipulate others in order to gain utility and avoid conflict [3]. Deceiving others is now an integral part of our behavior, from innocuous every-day (i.e. white) lies [4] to more elaborate cons, for example lonely hearts swindles [5].

Deceptiveness has been linked to intra- and intersexual competition, risk preferences and gender differences [6]. Ancestral human male reproductive fitness was constrained by access to females, and female fitness by factors related to providing for offspring survival [7]. Access to females and reproduction is a resource for which males engage in risky competition against one another [8], and in costly signaling directed towards females [9]. In modern men, this competition is reflected in deception to obtain status and influence, which indirectly result in better access to women [10]. Specifically, males are more likely to lie to secure a monetary benefit [11, 12], whereas females are generally seen as more cooperative and less exploitative [13]; although not all researchers agree with this [14]. The male competitive behavior also differs depending on the gender of their opponent [15]. In particular, male deception in intersexual competition (towards females) seems to be linked to status signaling [16, 17].

Gender stereotyping is present across contexts. For example, female trivia game contestants are often expected to be weaker competitors than their male counterparts [18], and car dealers will frequently quote higher prices to female as opposed to male customers [19]. In addition, females are perceived to be more easily misled and deceived when it comes to negotiations [20].

These findings extend to game theory, where Holm [21] demonstrated that irrespective of gender of the initiator, females would receive low-ball offers in a resource allocation task (i.e. they would be “hawkishly preyed upon”). Females would also be more likely recipients of attempts to deceive in a bluffing game [22]. Similarly, in the *ultimatum* game, both females and males were likely to choose a higher minimum acceptable offer when facing a female opponent [23, 24]; offers made to men were higher than offers made to women [23, 25], and women were more likely than men to accept lower offers [25]. Evidence thus suggests that both men and women display gender-dependent behavior in the context of competitive economic games involving incentives for selfishness and deception.

In contrast, in two-player *trust* games—where one player first sends to the other some amount of initial resources, which are then multiplied by a factor and distributed at the receiver’s discretion among the two players—participants have been found to send more resources to opposite gender partners than to same gender ones [26]. Males were also more cooperative towards females than males, especially when the females were smiling [27]. It is possible females receive more cooperative offers in trust games because they seem more trustworthy, but are defected against or “betrayed” in games involving deception and/or competition because they seem “weaker” or less dominant than males. Evidence suggests that characteristics such as “warm”, “trustworthy”, “friendly”, and “weak” are more often associated with females than males [28]. Moreover, similar patterns of results (i.e., females being offered more resources than males) have *not* been observed in studies using the *dictator* game, which is comparable to trust games but lacks the elements of *trust* and *reciprocation*: the initial resources are distributed between the two players at the *sender’s* (the “dictator’s”) discretion, and the receiver has no active role (see [29] for a review).

However, economic games are designed for laboratory use—results from studies employing them may not be generalizable or predictive of behavior in more naturalistic environments. Moreover, economic laboratory experiments typically have a very self-selected sample of participants (e.g., high-achieving students who have a tendency to want to please the experimenters; [30]). In economic games, such participant characteristics have a significant impact on behavior in terms of altruism, fairness and resource allocation. Thus, it is difficult to estimate the real life economic consequences of the decisions made in these games.

Unlike traditional economic games, *poker* is played frequently by more than a hundred million people worldwide—a card game where deception is the *norm*: no apparent ethical or social pressure prevents players from deceiving in poker. Game-theoretically, deception is also *necessary* to increase chances of winning [31]. The most common form of deception in poker is *bluffing*, which refers to betting or raising (showing strength) with a weak holding to make the opponent fold (give up). In other words, bluffing is misrepresenting the strength of one’s poker hand to trick opponents into believing the hand is (much) stronger than it actually is.

Poker is also a competitive, male-dominated game surrounded by strong gender stereotypes [32], and thus offers an excellent platform to study the social aspects of strategic deception. Male poker players often view female opponents as novices who are unwilling to take risks and easily intimidated [33]. Many also feel that females do not belong at the poker table, which is a place for “gutsy bluffs and betting warfare” [34]. Arguably, the stereotypical good (“strong”) poker player is aggressive, masculine, and fearless—hence the colloquial saying: “Don’t fold like a girl!”.

Poker is most frequently played *online*, where gender can be conveyed via *avatars*—graphical representations of players at a virtual table. The use of avatars in online poker is pervasive, and typically the avatar genders are clearly depicted [35]. Given how common bluffing is in poker, even small gender effects in players’ bluffing propensity might have highly significant monetary consequences especially for online poker players. Bluffing decisions quickly accumulate and become a substantial source of either wins or losses. Marginal changes in bluffing frequency might translate to thousands of dollars won or lost within weeks, or even days: Many active online poker players have been dealt millions of poker hands, and some are even able to play on 24 virtual tables simultaneously [36].

We hypothesized that players bluff more frequently at online poker tables with primarily female avatar opponents than at tables with primarily male avatar opponents. More importantly, we aimed to demonstrate that online poker is a useful tool in psychology, potentially offering new insights to the literature on deception, social psychology, and poker itself.

## Method

### Participants

An online survey created with Qualtrics (www.qualtrics.com) in English was advertised on various international online poker web-forums. Five hundred and fifty-eight participants completed the survey. Small effect sizes have been reported in previous online poker studies, calling for large sample sizes to increase statistical power [37]. Our data collection stopping rule was 180 participants per condition. However, based on *a priori* data exclusion criteria, we expected a final sample size of 160–170 participants per condition. Fifty-six participants were omitted due to insufficient skills in written English (skills not reported as “very good or better”). The final sample size was 502 (N = 502; 435 males, 36 females, 31 unreported; *M*_age_ = 29.99; *SD*_age_ = 8.72; age ranged from 16 to 67). These demographics are consistent with previous studies sampling poker playing populations [37]. Participants were offered the possibility of taking part in a draw of five separate $50 Amazon.com gift coupons. This study was approved by the Newcastle University ethics committee.

### Procedure

Upon opening the questionnaire, participants gave informed consent and were randomly assigned to one of three conditions in a between-subjects design. Participants first completed the covariate measure of poker experience and three other measures unrelated to current aims, followed by the dependent measure/variable (bluffing tasks), manipulation checks, and demographics.

Participants made *bet / do not bet* (i.e., bluff / do not bluff) decisions in four simulated poker tasks, in which they were “sitting” at an online poker table with four opponents represented by avatars and the names “Opponent 1–4”. The between-subjects factor “Avatar Balance” had three levels referring to the total number of female and/or male opponents (avatars) at the table: 1) all female, 2) all male, and 3) two male and two female (“mixed”) avatars (cf. S1 Supporting Information p. 1 for pictures). The tasks were otherwise identical across these three conditions and presented in random order.

### Materials

#### Avatar Creation and Pretest

Facegen Modeller v3.5 (Singular Inversions, www.facegen.com) was used to create male and female faces of similar age (around 30) with neutral expressions and various hairstyles. Facegen Modeller is frequently used to create realistic human faces in psychological research [38]. In total, 18 male and 18 female faces were created for pretesting. An online questionnaire was distributed via Amazon Mechanical Turk, asking 100 participants to evaluate the faces in a random order, one per page, on Likert 1 (“not at all”)– 7 (“very”) scales on the following attributes: friendliness, trustworthiness, warmth, competence, likability, dominance, threat, and attractiveness. To obtain avatars differing mainly with respect to their perceived gender, we selected four female and four male faces that were the “most neutral” with respect to all evaluated attributes (mean values ~4).

#### Covariate (Poker Experience Scale [PES])

PES has been shown to predict mathematical accuracy in poker decision-making and used in several studies to measure players' poker skill and knowledge [37, 39]. Because bluffing also involves skill, it is likely that poker experience influences bluffing behavior. Thus, we included PES as a covariate. The scale consisted of three 11-point and one 10-point Likert items, which measure 1) amount of years played, 2) average level of stakes played, 3) amount of poker hands played, and 4) level of self-perceived “professionalism” in poker (cf. S1 Supporting Information p. 4 for item details). The 4-item scale (*M* = 5.23, *SD* = 2.12, range = 1–10) was normally distributed and had a satisfactory inter-item reliability (Cronbach's alpha = .80). Higher scores indicate higher level of poker experience and skill.

#### Dependent Variable (Bluffing Measure)

Participants undertook four simulated online poker tasks involving the most popular poker variant, No Limit Texas Hold’em (NLHE). The task was in a cash-game format, i.e., not a tournament format: the chips at play represented cash (hypothetical US dollars), and not tournament points. Participants first read detailed task instructions and indicated having understood them (by self-report; cf. S1 Supporting Information p. 2). It is worth noting that studying NLHE experimentally is very difficult due to its complexity, but with care, this complexity can be reduced by evaluating specific aspects of the game without losing ecological validity. Below, we first briefly introduce the rules of NLHE, and then explain how our bluffing tasks were constructed.

In NHLE, two cards are first dealt face down to each player, followed by a round of betting (period called *pre-flop*). Then, five community cards that can be used by all players are placed face-up on the table. The first three community cards are called the *flop*, and the last two cards the *turn* and *river*, and there is a round of betting after each. This period of play beginning with pre-flop and ending at latest on the river is called a *hand*.

During a NLHE hand, players have multiple opportunities to bluff during pre-flop, on the flop, turn, and river. Bluffs on the river almost always end the hand (they are either called down or folded against; a bluff on the river might get *raised*, but in these cases the bluffer almost always gives up and folds), whereas other bluffs are sometimes followed by another round of betting (if a bluff on the turn is called, another round of betting takes place on the river). For simplicity, participants in our experiment bluffed only on the river.

In order to make an informed bluffing decision on the river, it is highly important for players to know what the opponents’ preceding betting actions were. A realistic bluffing task needs to give participants this information. We presented each task as an animated sequence of “automated” betting actions beginning pre-flop, and ending on the river, upon which participants decided to either *bet* or *check* (not bet). Participants could not influence these automated actions. We consulted professional poker players to make sure the game scenarios were as realistic as possible for NLHE with five players, and emulated as closely as possible the graphical outlook of the tables used by the most popular online poker site, www.pokerstars.net. See http://www.comp.lancs.ac.uk/~yanj2/poker/ for the tasks exactly as they were presented to participants (the tasks are presented as animated frame-by-frame sequences of betting actions, and viewing each task takes about 80 seconds).

The opponents (avatars) and the participant were “sitting” in the same table position across the four tasks. Participants made one decision against each opponent, and each decision was made in a *heads-up* situation (one versus one; the other three opponents had folded their cards and were not “in the hand”).

If the participant decided to bet, s/he first indicated the amount to bet (hypothetical US dollars) and then whether or not the bet was a *bluff* (“Was your bet a bluff? Yes/No/I don’t know”). Only the bets that participants reported being bluffs were analyzed as actual bluffing decisions. Of all the betting decisions made by all the participants, 97% were reported to be bluffs. This indicates we were successful in creating the tasks in a way that the only sensible reason to bet would be to bluff. The DV “Average Bluffing Frequency” was calculated for each participant as the total number of bluffing decisions (maximum = 4, minimum = 0) divided by four (*M* = 0.45, *SD* = 0.23).

## Results

### Manipulation Checks

After the poker tasks, participants answered the following question on a Likert scale 1 (“completely disagree”)– 7 (“completely agree”): “The gender of the opponents' avatars influenced my decisions”. Participants were then presented with pictures of the poker task avatars, in a randomized order and one per page, and asked to evaluate them on the same attributes that were used in the pretest (see S1 Supporting Information p. 11). Participants were also asked to indicate the gender of the avatar picture (“Is this person: Male/Female”).

The majority of participants disagreed with the statement “The gender of the opponents’ avatars influenced my decisions” (*M* = 1.39, *SD* = 0.97). The average avatar gender recognition rates were 93.5%, 99.7%, and 98.7% in the “all female”, “all male”, and “mixed” avatar groups, respectively.

### Main Analyses

**Fig 1:**
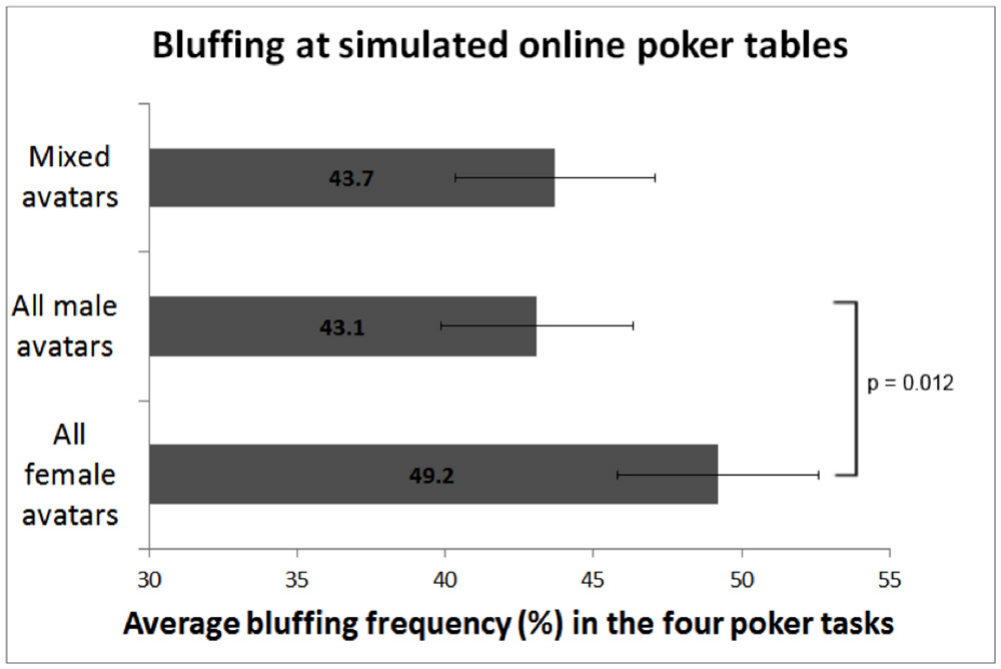
Average bluffing frequency (%) in the four poker tasks. Results are presented separately for three experimental conditions: poker table with 1) gender mixed (two female and two male), 2) all male, and 3) all female avatar opponents. The model is adjusted for Poker Experience Scale. Planned contrast between “all male avatars” and “all female avatars” groups is significant: B = 0.061, *F*(1, 498) = 6.41, 95% CI [0.01, 0.11], *p* = .012. Error bars represent 95% confidence intervals.

A one-way ANCOVA was run with Average Bluffing Frequency as the DV, Avatar Balance as a between-subjects factor, and Poker Experience Scale as a covariate. There was a significant main effect of Avatar Gender on Average Bluffing Frequency (*F*(2, 498) = 3.83, *p* = .022, par. η² = 0.015). Planned contrast analysis revealed that participants bluffed more frequently at tables with all female avatars than with all male avatars (B = 0.061, *F*(1, 498) = 6.41, 95% CI [0.01, 0.11], *p* = .012). Participants also bluffed more frequently at tables with all female avatars than at gender mixed tables (B = 0.055, *F*(1, 498) = 5.10, 95% CI [0.007, 0.10], *p* = .024). Participants with more poker experience bluffed more frequently (B = 0.039, *F*(1, 498) = 69.3, 95% CI [0.03, 0.048], *p* < .001, par. η² = 0.122). See Fig 1 and Table 1 for model statistics.

**Table 1:** ANCOVA statistics. Average Bluffing Frequency is the dependent variable.

Factor	*df*	*F*	*p*	par. η²
Avatar Balance	2	3.83	.022	015
Poker Experience Scale	1	69.3	< .001	.122

Model statistics: *F*(3, 498) = 24.5, *p* < .001, adj. *R*^2^ = 0.123). The factor “Avatar Balance” has three levels referring to the number of female and/or male opponents (avatars) at the virtual online poker table: 1) gender mixed (two female and two male), 2) all male, and 3) all female avatars.

### Supplementary Analyses

Supplementary analyses were performed by entering gender, age, education and income as covariates (cf. S1 Supporting Information pp. 9–10 for statistics on education and income) and omitting data from the participants who did not recognize the avatar opponent genders with 100% accuracy or had missing demographic data. Males bluffed more frequently than females (B = 0.129, *F*(1, 427) = 10.1, 95% CI [0.05, 0.21], *p* = .002). The younger the participants were, the more frequently they bluffed (B = -0.003, *F*(1, 427) = 6.515, 95% CI [-0.006, -0.001], *p =* .011). The main findings were robust to adding the demographic controls into the model. The current study included also measures (questionnaire scales) unrelated to the current aims and hypotheses. The main results were robust to including these measures into the models as covariates. The results concerning these measures have been reported in [40]. Consult S1 Supporting Information pp. 5–6 for more details, including a data transparency table.

As a *post hoc* analysis, we also contrasted the”all female” group with the combined mean of the”all male” and”gender mixed” groups (”all female” vs.”gender mixed” +”all male”). This contrast was statistically highly significant (B = 0.12, *F*(1, 498) = 7.59, 95% CI [0.03, 0.20], *p* = .006). Given the gender imbalance in our sample, we re-ran all our analyses by excluding all female participants. This did not significantly affect the results.

### Expected Monetary Value of Bluffing

In order to evaluate the monetary implications of the observed 6.1% difference in bluffing frequency (cf. Fig 1), we performed *post hoc* expected value calculations. These were made possible by having data on the participants’ bluffing frequency and their average bluff sizes. Specifically, we calculated the expected value of calling the bluffs made by the participants—or, in other words, the monetary implications for the hypothetical *opponents* in our experiment. Below, for clarity, we will refer to the hypothetical opponent as “Player 1” and the participant as “Player 2”.

Across the four tasks, the average size of the pot (amount of contended money) on the river was $301.7, and the average bluff sizes were $212 (*SD* = 91.9) and $210 (*SD* = 93.6) at the all male and all female tables, respectively. Player 1 needs to call these amounts for a potential gain (equaling the size of the pot before calling) of $212 + $301.7 = $513.7 (all male table) or $210 + $301.7 = $511.7 (all female table). For simplicity, we make the following assumptions:

1. Generally, a bet on river (see Glossary in S1 Supporting Information p. 3) in online poker is either made as a bluff or “for value” (i.e. in hopes of getting called by a worse hand)
2. In actual online poker, in similar “river positions” as those used in our experiment, the player who is betting is typically equally likely to have a strong hand as a weak one, and very unlikely to have a hand of medium strength. Therefore, we assume Player 2 will have a strong hand (i.e. a winning hand) 50% of the time, and a weak hand (i.e. a losing hand) 50% of the time. Although this assumption is a simplification, it is based on the feedback from two professional poker players we have consulted. The exact distribution of hand strengths in similar river positions is impossible to ascertain, given the imperfect information nature of the game
3. In similar river positions as those used in our experiment, strong hands will always bet “for value” (i.e. they will never “check”) and the bet sizes will be similar to those of the bluffs

**Fig 2:**
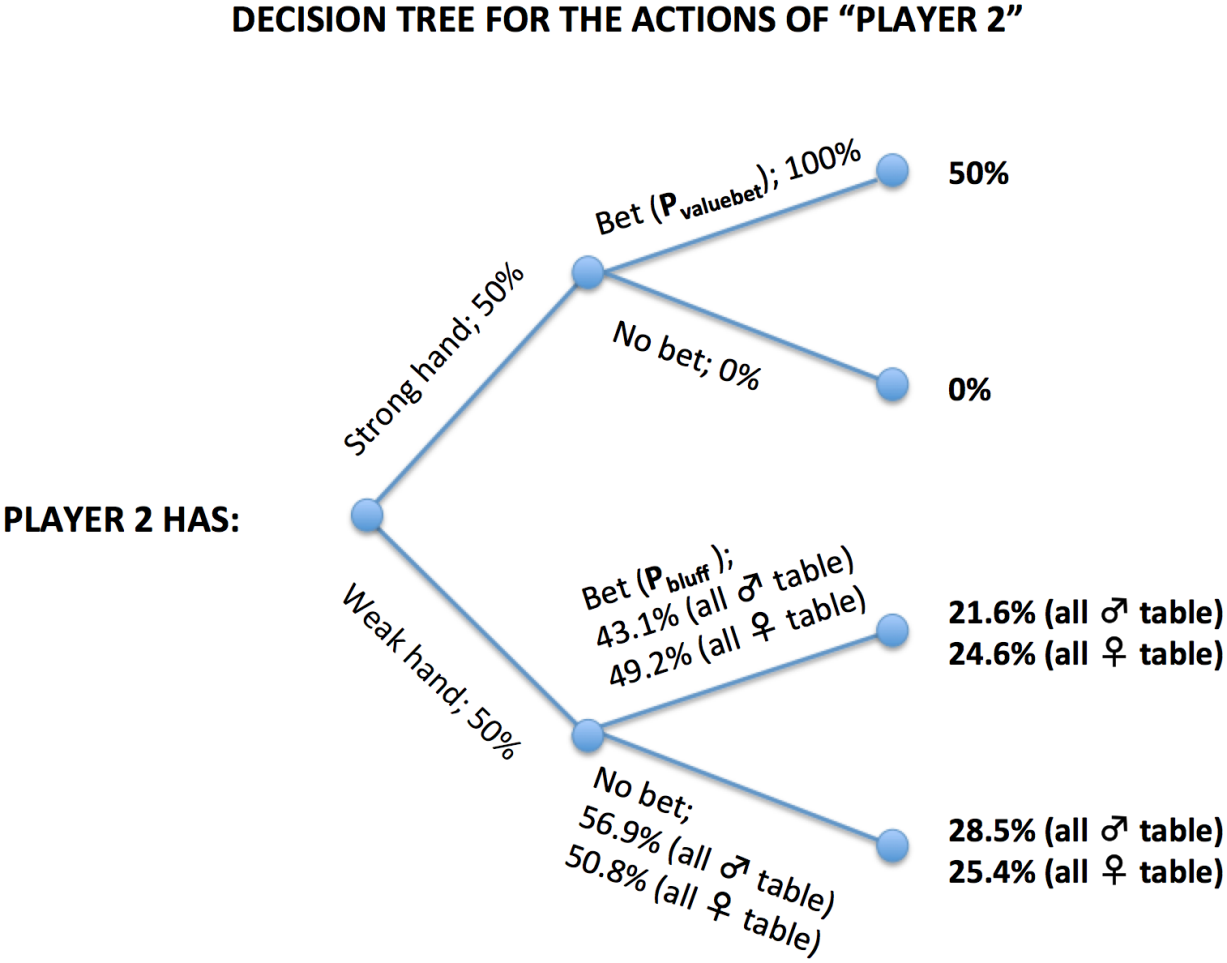
Decision tree for the actions of player 2. The probabilities for “Strong hand” and “Weak hand” are derived from Assumption 2, and the probability for **P**_**valuebet**_ is derived from Assumption 3. The probabilities for **P**_**bluff**_ are obtained from our experimental data. “All ♂ table” and “All ♀ table” refer to the experimental conditions “all male table” and “all female table”, respectively.

Given these assumptions, we can extrapolate the results from the current experiment to actual online poker. Player 2 will have a strong/winning hand 50% of the time, of which s/he will bet for value ***P***_**valuebet**_ = 1 = 100% of the time; and a weak/losing hand 50% of the time, of which s/he will bet (i.e. bluff) ***P***_**bluff**_ of the time. We obtain the values of ***P***_**bluff**_ from our experimental observations: The average bluffing frequencies were 43.1% (at all male tables) and 49.2% (at all female tables; cf. Fig 1). Note that every bet made with a weak hand is by definition a bluff, because all bets in our experiment were made with weak hands, and only bets that were considered to be bluffs were included in the analyses. Thus, following the Bayes’ theorem notation, the probability of Player 2 having a weak/losing hand given s/he’s betting (i.e. the posterior probability of Player 2 bluffing; see Fig 2 for a decision tree illustration), is:

$$\begin{array}{l} P\left(A|B\right)=\;P(“Player\;2\;has\;a\;weak\;hand”\;|\;“Player\;2\;bets”)\\ =\frac{P\left(A\cap B\right)}{P\left(B\right)}=\frac{P\left(B|A\right)\times P\left(A\right)}{P\left(B\right)}\\ =\frac{\;P(“Player\;2\;bets”\;|\;“Player\;2\;has\;a\;weak\;hand”)\;\times \;P\left(“Player\;2\;has\;a\;weak\;hand”\right)}{P\left(“Player\;2\;bets”\right)}\\ =\;\frac{P_{bluff}\;\times \;0.5}{P_{valuebet}\;\times \;0.5+\;P_{bluff}\times \;0.5}\;\;=\;\;\frac{P_{bluff}}{P_{valuebet}\;+\;P_{bluff}} \end{array}$$

At all male tables this yields:

$$P\left(A|B\right)=\;\frac{0.431}{1+0.431}\approx 0.3012$$

At all female tables:

$$P\left(A|B\right)=\frac{0.492}{1+0.492}\approx 0.33$$

Because **P(A|B)** is the posterior probability of Player 2 bluffing, it is equal to the probability of Player 1 winning by calling. Thus, the expected value of calling is:

P(A|B) × [Size of the pot before calling] – (1 – P(A|B)) × [Size of the bet]

At all male tables, this yields:

0.3012 × $513.7 – 0.6988 × $212≈$6.53

At all female tables:

0.33 × $511.7 – 0.67 × $210≈$28.16

Thus, we estimate that calling the bets made by Player 2 would be **$28.16 − $6.53 = $21.63** more profitable *per bet* when done at all female tables, compared with all male tables.

## Discussion

We evaluated gender effects in online poker bluffing. Participants were more likely to bluff with opponents represented by female avatars than with male or gender-mixed avatars. Supplementary analyses revealed that male participants also generally bluffed more frequently than females.

These results are consistent with previous studies evaluating two distinct aspects of gender effects in competition and deception in economic games. Firstly, Holm [21, 22] and Solnick [23] found that knowledge of co-player gender influenced both male and female participants' behavior: both behaved aggressively when they believed their co-player was female. Secondly, our results tentatively support Dreber and Johannesson [11] and Erat and Gneezy [12], who found that males were more likely than females to deceive for a monetary benefit.

Poker highlights gender stereotypes and intersexual competition. Often the callous purpose of the game is to win as much money (and fame) as possible at other players' expense. Consistent with Holm [21], participants' bluffing behavior in the current experiment can be viewed as “hawkish”; female avatars might be regarded as opponents who are unlikely to “fight back”, and easy targets for bluffing. Thus, female players presumably get “preyed upon” in competitive games involving deception due to stereotypes in opposition with a good, “strong” (male) player. This interpretation is also consistent with Kray et al. [20], who found that women are deceived more often than men in negotiations due to their lower perceived competence. Taken together, these results help explain why similar gender-conditioned behavior has not been prominently observed in the context of *trust* games, where success depends on the “benevolence” (and not the “gullibility”) of one's co-player [26, 27].

We expected that bluffing frequency would increase in the order of “all male” < “gender mixed” < “all female” tables (groups). However, there was no significant difference between the “all male” and “gender mixed” groups. If a player decides to bluff a female avatar at a gender mixed table, the decision is made in the *presence* of male avatars. Sensible players should be cautious not only of their current “heads-up” opponent but also of the other players at the table, because future decisions probably need to be made against them as well. Ostensibly, at an “all female” table (unlike at “all male” and “gender mixed” tables) players do not need to worry about having to face “strong male players” and being exploited by them. This hypothesis is sensible, since the participants made decisions against *every* opponent; at the “gender mixed table”, the male opponents were not “merely observers”.

Another possible explanation relates to lack of salience in gender information in the current experiment. Evidence suggests that gender salience affects the strength of gender-conditioned behavior. For example, Datta Gupta et al. [15] observed that participants behaved more competitively against female co-players than against male ones, but only when they knew their co-player's (female) nickname actually belonged to a female—i.e., when they were presented with “strong” as opposed to “weak” gender information. In our experiment, opponent nicknames were gender-neutral (“opponent 1–4”). Gender salience could be increased by employing gender-specific nicknames, or by increasing the attractiveness of the avatars, which might elicit stronger effects.

On the other hand, an interesting venue for future work is to evaluate ways for female avatars to signal their competence to reduce being deceived. Although competence is typically associated with masculine features [38], there are probably ways to decrease the perceived “bluffability” of female avatars without having to swap gender—for example by having female avatars with shorter hair (or no hair), or angrier expressions.

Participants were not explicitly told that a female avatar “belongs” to a female player, because in online poker players rarely know their opponents' genders with certainty. Despite uncertainty about player genders, the perceived gender of avatars has been shown to influence behavior online. For example, female online poker avatars were observed receiving more inappropriate chat comments than their male counterparts [41]. Hussain and Griffiths [42] found that some females believed they can avoid online harassment by assuming a male avatar in role-playing games. Avatars alone can act as gender-cues and predispose players to behave in a gender-stereotyping manner—although increasing the salience of gender information can strengthen this effect.

It is noteworthy that most participants did not believe the avatar genders influenced their decisions, suggesting that the observed effect on bluffing was *implicit*. For many players, especially experienced ones, bluffing depends heavily on the opponents’ previous betting “patterns”. For example, an opponent who first bets a large amount pre-flop (cf. Glossary in S1 Supporting Information p. 3) but abruptly stops betting afterwards might seem suspicious due to an “inconsistent” betting style. Putatively, the participants in the current study paid closer attention to information about the opponents’ betting patterns than their (avatar) genders. Our results nonetheless demonstrate that “weak” opponent gender information is sufficient to influence behavior, even when little conscious attention is paid to the avatars.

In terms of limitations, our study was Internet-based, and thus we were unable to control for possible distractions. In line with the current results, previous studies [36, 37, 39] have demonstrated that in online poker communities, at least 90% of the players are male. Because females are underrepresented among poker players, they might be a selected group of players who are more competitive than non-poker playing females. Given the significant gender imbalance in our sample, the results on gender differences should be viewed with caution and considered as tentative, as they might not be generalizable. In terms of our main results, we are limited to the conclusion that *at least* male players (but not necessarily females) bluff more frequently at online poker tables with female avatar opponents than at tables with male ones. Nonetheless, our sample was diverse in terms of education, income and age, which is often not the case in psychological studies sampling student populations within a single university. Finally, participants did not play an actual poker game or wage their own money, which reduces ecological validity. However, because we used realistic visual poker scenarios emulating the most popular online poker site (www.pokerstars.net), our setting was more ecologically valid than others previously used successfully in online poker research [37]. We also received positive comments from some of our participants about the tasks, saying our poker scenarios felt “surprisingly” similar to actual online poker decisions.

In conclusion, the current study demonstrated gender effects in online poker bluffing, which arguably stem from gender-stereotyped beliefs about male and female players’ competitive characteristics. Importantly, online poker is an environment where the features of players’ avatars likely have significant monetary consequences. In online poker, multiple betting decisions involving real money are made within short periods of time, and many active players have made millions of betting decisions in total [36]. Thus, if avatar gender can even marginally influence decision-making, this effect will rapidly accumulate. In a similar vein, casinos typically exclude from their premises anyone who is able to obtain a marginal edge over them (e.g., a 0.5% edge in blackjack; [43]). An estimated difference of 6% in bluffing frequency, as we observed, is thus highly significant. This is further supported by our expected monetary value calculations, which suggested there is a difference of about $22 per bet in bluffing profitability when playing at online poker tables comprising of all female avatars as opposed to all male ones.

In general, poker has been studied only occasionally in psychology [44], and poker studies focusing on deception are particularly scarce. We advocate that future research can gain much from employing poker to study the multifaceted phenomenon of deception. For example, in addition to bluffing, there are other forms of deception in poker. One of these is *slow-playing* (or *trapping*), which is roughly the opposite of bluffing: betting weakly or not at all with a very strong hand to “lure” the opponent into betting or raising with a hand they would normally not play with (luring someone into a “trap”). Furthermore, a player who is known for bluffing might play with a very strong hand, making it seem *as if* s/he were just bluffing again. This is an example of “deceiving by telling the truth” and adds another layer to ways in which people might deceive and manipulate each other in poker.

These various types of deception can be conveniently investigated by using poker as an ecologically valid tool, but probably not otherwise. Such studies will likely reveal new insights on deception in poker, a popular and pervasive game, and on the psychology of deception in general. Therefore, poker is a useful addition to the common toolbox for psychology research.

## Supporting Information

S1 Supporting Information Multiple figures and tables, stimulus materials, and other additional information (statistical analyses, other covariates unrelated to current aims, data transparency table, and a glossary of poker terminology) in a single file. (DOCX)
