# Supplementary material for: "To Bluff like a Man or Fold like a Girl?" – Gender Biased Deceptive Behavior in Online Poker
Source: PLoS One. 2016 Jul 6;11(7):e0157838. doi: 10.1371/journal.pone.0157838 (PMC4934693; doi:10.1371/journal.pone.0157838)
Supplement: S1 Supporting Information — (DOCX) [file pone.0157838.s001.docx]

**Supporting Information**

**Avatar Pictures**


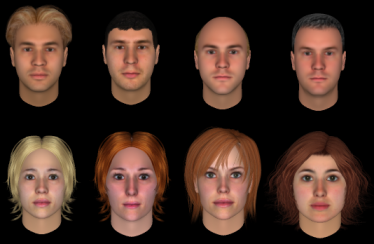


**Figure 1.** Male (top) and female (bottom) avatars used in the experiment.


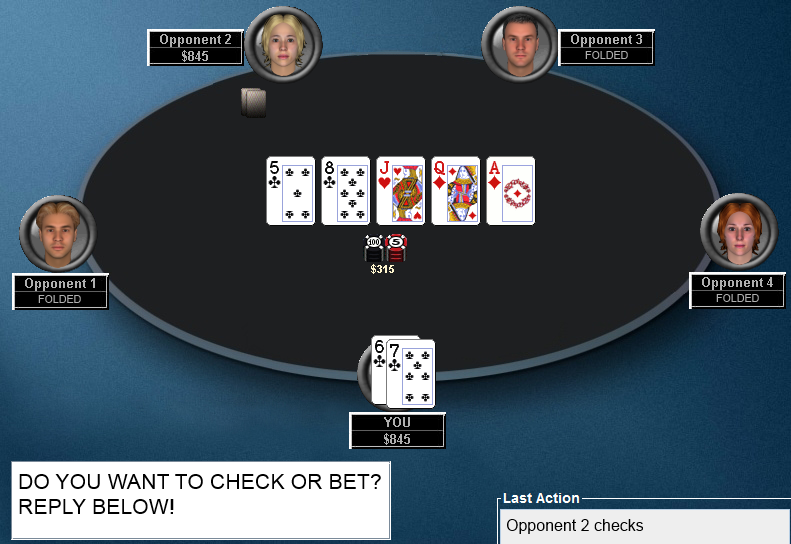


**Figure 2.** Example picture of the bluffing task from the “mixed” group (gender mixed avatars). For animated betting sequences of all tasks, see http://www.comp.lancs.ac.uk/~yanj2/poker/

**Bluffing Task Instructions**

The following instructions were shown to participants:

**“**Please read these instructions carefully!

On the next pages, you will be shown four visual online poker scenarios, one per page. The game is No Limit Texas Hold’em, cash/ring game (not a tournament!), played at a table with 4 opponents and yourself (5 players in total). The opponents are represented by avatars.

The blinds are $5 and $10, and each player has $1000 at the beginning of each round. Four rounds (or “hands”) will be played in total.

Each round is automatically “played through” as frame-by-frame animated actions made by the players (including you!) at the table. **Please pay attention during this time, as you will not be shown the actions again!**

**You cannot influence these actions**, and you have to wait until the animation is finished – for each round, this will take about 80 seconds.

The animation finishes before the last possible action (“on the river”), and you will be asked to make a decision to either **1) CHECK, or 2) BET**. If you decide to BET, you will also be asked to write down the size (in numbers) of your bet.

Make your decision based only on the information provided on this page, and on the animations themselves. Play as you would normally online (even if you don't normally play with the current level of stakes), against otherwise "unknown" opponents.

Click on the arrow ">>" to continue to the first scenario!

Please indicate below that you have understood the above instructions.**”**

**Glossary**

**Poker Hand** Depending on the context, a Hold'em poker hand may refer to either **1)** a

single round of game play; the period beginning when cards are dealt and ending with the showdown (revealing of players’ cards and deciding the winner of a given hand), **2)** the two cards dealt to each player at the beginning of each round of game play (also referred to as the starting hand or hole cards), or **3)** the best five-card combination that can be formed using the hole cards and the community cards. Use of the term “hand” in this article refers to definition **1**.

**NLHE** No Limit Texas Hold'em – A popular variation of the standard game of poker. Any game of poker is a card game involving betting whereby the winner is determined based on the ranking of their cards. NLHE consists of two cards being dealt face down to each player, and then five community cards – cards that can be used by all players – being placed face-up. Players have the option to **check**, **call**, **bet**, **raise**, or **fold** either prior to the flop (**pre-flop**), on the **flop**, on the **turn**, and on the **river**.

**Blinds** Forced bets (small and big blind) that are placed into the **pot** by players before play begins

**Button** The **(dealer) button** is a marker used to indicate the player who acts last on that deal.

**Pre-flop** The period beginning with the dealing of cards and ending with the **flop** in Hold'em poker

**Flop**  The first three cards dealt face-up to the board in Hold'em poker

**Turn**  The fourth card dealt face-up to the board in Hold'em poker

**River**  The fifth and final card dealt face-up to the board in Hold'em poker

**Pot** Sum of money (or chips) that players have waged during a single **hand** (definition **1**) of game play

**Bet** To wager an initial amount of money

**Fold** To discard one’s cards and give up playing during the current **hand** (definition **1**), thereby forfeiting interest in the current **pot**

**Check** Declining to make a **bet**, but retaining the right to **call** or **raise** bets or raises made by subsequent players

**Call**  Matching a **bet** or a **raise** made by another player

**Raise** Increasing the size of a **bet** required to stay in the pot, forcing all subsequent players to call the new amount (or raise more) if they wish to remain in

**Details on Poker Experience Scale (PES)**

To reduce previously observed skewness in PES, we modified it slightly for the current study. The modified PES included the following items. *How many years have you played poker?* (1 = Less than 0.5 [6 months]; 11 = More than 15); *At what level of stakes do you usually play?* (1 = No real money stakes, just for fun; 11 = Above NL600, PLO600, SNG500, MTT500); *What is the rough estimate of how many poker hands you have played during your life?* (1 = 0–10 000; 11 = more than 5 million); and *Do you consider yourself to be a professional poker player?* (1 = Definitely not a [full time] professional poker player; 10 = Definitely a [full time] professional poker player). The stakes abbreviations are NL = No Limit (Hold’em); PLO = Pot Limit Omaha; SNG = Sit’n’Go (i.e., single table tournament); MTT = Multi Table Tournament. The number following the abbreviation refers to the minimum buy-in (in US dollars) to play.

**Other Study Covariates**

**Machiavellian Personality Scale.** In this 16-item scale, Machiavellianism is conceptualized as individual propensity to distrust others, engage in amoral manipulation, seek control over others and status for oneself. The scale consists of 16 items and 4 subscales (example items in brackets): Amorality (“I would cheat if there was a low chance of getting caught”), Desire for Control (“I enjoy having control over other people”), Desire for Status (“Status is a good sign of success in life”), and Distrust of Others (“People are only motivated by personal gain”). All items are anchored from 1 (“Completely disagree”) to 7 (“Completely agree”).

*Source:* Dahling, J. J., Whitaker, B. G., & Levy, P. E. (2009). The development and validation of a new Machiavellianism scale. *Journal of management*, 35(2), 219-257.

**Masculinity Trait Index.** This 10-item scale measures one’s own self-perceived "masculine" characteristics. Example items are: “I am competitive” and “I have leadership abilities”. All items are anchored from 1 (“Completely disagree”) to 7 (“Completely agree”). Higher scores indicate higher self-perceived masculinity.

*Source:* Stern, B. B., Barak, B., & Gould, S. J. (1987). Sexual identity scale: a new self-assessment measure. *Sex Roles*, *17*(9-10), 503-519.

**Sensitivity to Losses.** This 11-item scale measures the extent to which poker players experience negative emotions (e.g., feelings of unfairness, anger and frustration) elicited by poker losses. Example items are: “I feel losing is unfair” and “When I lose, I feel anger”. All items are anchored from 1 (“Completely disagree”) to 7 (“Completely agree”). Higher scores indicate a higher tendency to experience negative emotions of, e.g., unfairness, anger and frustration elicited by losses.

*Source:* Palomäki, J., Laakasuo, M., & Salmela, M. (2014). Losing more by losing it: Poker experience, sensitivity to losses and tilting severity. *Journal of Gambling Studies*, *30*(1), 187-200.

**Sensitivity to Slow-play.** This scale was developed by the authors. It measures the extent to which individuals experience negative emotions in poker when they lose after being slow-played against. In other words, it measures emotional sensitivity to “getting slow-played”. The scale consists of 3 items, all of which are anchored from 1 (“Completely disagree”) to 7 (“Completely agree”). The items are: 1) “*If I get slow-played and lose, I feel my opponent is playing ‘dirty’*”; 2) “*If I get slow-played and lose, I feel angry*”; 3) “*If I get slow-played and lose, I feel exploited*“.

**Data transparency**

The data reported in the current manuscript were collected as part of a larger data collection (at a single point in time). Findings from the data collection have been reported in two separate manuscripts, one being the current one, and the other being referenced below. The second manuscript focuses primarily on variables “Machivellian Personality Scale”, “Sensitivity to Slow-play”, “Bluffing Frequency” and “Average Bluffsize”, and in part on variable “Masculinity Trait Index”. The table below displays where each data variable appears in these manuscripts. All analyses in both manuscripts were controlled for all variables in the full dataset, and there were no confounding factors. These two manuscripts are independent of one another, and have unrelated aims.

| **Variables in the complete dataset** | Current manuscript | Machiavelli as a poker mate |
| --- | --- | --- |
| Avatar Gender Balance | X |  |
| Bluffing Frequency | X | X |
| Average Bluffsize |  | X |
| Poker Experience Scale | X |  |
| Machivellian Personality Scale |  | X |
| Sensitivity to Slow-play |  | X |
| Sensitivity to Losses |  | X |
| Masculinity Trait Index |  | X (in part) |

Palomäki, J., Yan, J., & Laakasuo, M. (2016). Machiavelli as a poker mate—A naturalistic behavioural study on strategic deception. *Personality and Individual Differences*, *98*, 266-271.

**Textual Descriptions of the Poker Tasks**

Another way to present the bluffing tasks is to use textual descriptions of the betting sequences, (as done by Palomäki et al., 2013a and Laakasuo et al., 2014) which are shown below. However, textual task descriptions are taxing to read, especially for inexperienced players, and do not visually resemble an actual online poker environment. By employing animated poker tasks, we evaded these issues.

**Task 1**

You are in the button position with **[6c 7c]** (six of clubs, seven of clubs), and the actions is folded to you (before the flop, the two players acting before you have folded). You “auto-bet” $25, the small blind folds, and the big blind calls. The pot is $55, and the game is “heads-up” (one versus one). The flop is **[5c 8c Jh]** (five of clubs, eight of clubs, jack of hearts), and the opponent checks. You “auto-bet” $40, and the opponent calls. The pot is $135. The turn is **[Qd]** (queen of diamonds), and the opponent checks. You “auto-bet” $90, and the opponent calls. The pot is $315. The river is **[Ad]** (ace of diamonds). The opponent checks.

The board is now **[5c 8c Jh][Qd][Ad]**, the pot is $315, and you are holding **[6c 7c]**. Do you check or bet?

**Task 2**

You are in the big blind position with **[8s 9s]** (eight of spades, nine of spades). Before your turn to act, three opponents call the big blind of $10, and the opponent in the small blind position raises to $30. You “auto-reraise” to $130. Three opponents fold, and the opponent in the small blind position calls. The pot is $290, and the game is “heads-up” (one versus one). The flop is **[2d Jd 6d]** (two of diamonds, jack of diamonds, six of diamonds), and the opponent checks. You “auto-check”. The turn is **[3d]** (three of diamonds), and the opponent checks. You “auto-check”. The river is **[Qh]** (queen of hearts), and the opponent checks.

The board is now **[2d Jd 6d][3d][Qh]**, the pot is $290, and you are holding **[8s 9s]**. Do you check or bet?

**Task 3**

You are in the button position with **[Ac 6s]** (ace of clubs, six of spades). Before your turn to act, two opponents call the big blind of $10. You “auto-raise” to $50, the opponent in the small blind calls, and the two other opponents (who called $10) fold. The pot is $130, and the game is “heads-up” (one versus one). The flop is **[Ks Tc Qh]** (king of spades, ten of clubs, queen of hearts)**,** and the opponent checks. You “auto-bet” $90, and the opponent calls. The pot is $310. The turn is **[2h]** (two of hearts), and the opponent checks. You “auto-check”. The river is **[9h]** (nine of hearts), and the opponent checks.

The board is now **[Ks Tc Qh][2h][9h]**, the pot is $310, and you are holding **[Ac 6s]**. Do you check or bet?

**Task 4**

You are in the button position with **[2s 2c]** (two of spades, two of clubs). Before your turn to act, the opponent who is first to act raises to $50, one opponent folds, and you “auto-call” the bet of $50. The opponents in the small and big blinds fold. The pot is $115, and the game is “heads-up” (one versus one). The flop is **[7h Th 9s]** (seven of hearts, ten of hearts, nine of spades), and the opponent checks. You “auto-bet” $90, and the opponent calls. The pot is $295. The turn is **[3h]** (three of hearts), and the opponent checks. You “auto-check”. The river is **[Jc]** (jack of clubs), and the opponent checks.

The board is now **[7h Th 9s][3h][Jc]**, the pot is $295, and you are holding **[2s 2c]**. Do you check or bet?

**Additional Statistical Analyses**

In our study, both education and annual income (in US $) were measured with Likert 1–7 scales. The lowest and highest anchors were 1 = “Elementary school”; 7 = “Scientific doctorate (e.g. PhD)”, and 1 = “<20 000”; 7 = “>100 000” for education and income, respectively. Of the participants in our sample, 22.9% had no college education, 27% had some college education but no degree, 28.9% held a bachelor's degree, and 21.3% held a master's degree or higher. On average, our participants reported an annual income between US$30.000 and $40.000 (ranging from below $20.000 [35.1%] to above $80.000 [11.2%]).

Table 1. ANCOVA statistics. Average Bluffing Frequency is the dependent variable. Data is omitted from the 67 participants who did not recognize the gender of the avatar opponents with 100 % accuracy or had missing data on demographics.

| **Factor** | *df* | *F* | *p* | par. η² |
| --- | --- | --- | --- | --- |
| Avatar Balance | 2 | 3.58 | 0.029 | 0.016 |
| Gender | 1 | 10.1 | 0.002 | 0.023 |
| Age | 1 | 6.52 | 0.011 | 0.015 |
| Education | 1 | 1.07 | 0.3 | 0.003 |
| Income | 1 | 0.32 | 0.57 | 0.001 |
| Poker Experience Scale | 1 | 46 | < 0.001 | 0.097 |

*Note.* Model statistics: *F*(7, 427) = 13.9, *p* < .001, adj. *R*^2^ = 0.172). Males bluffed more frequently than females (B = 0.129, *F*(1, 427) = 10.1, 95% CI [0.05, 0.21], *p* = .002). The younger the participants were, the more frequently they bluffed (B = -0.003, *F*(1, 427) = 6.515, 95 % CI [-0.006, -0.001]).


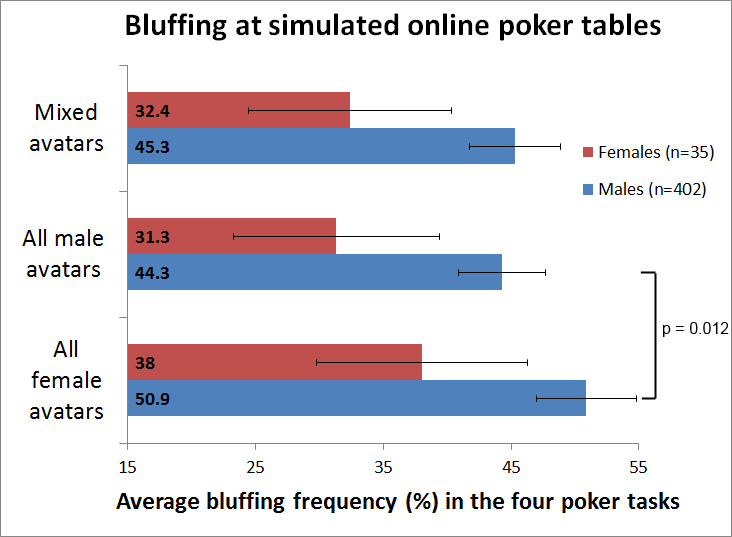


**Figure 3.** Average bluffing frequency (%) in the four poker tasks, presented separately for male and female participants, and for three experimental conditions: poker table with 1) gender mixed (two female and two male), 2) all male, and 3) all female avatar opponents. The model is controlled for age, education, income and Poker Experience Scale, and omitting the 67 participants who did not recognize the gender of the avatar opponents with 100 % accuracy or had missing data on demographics (see Manipulation Checks in main text). Planned contrast between “all male avatars” and “all female avatars” groups is significant: B = 0.067, *F*(1, 427) = 6.5, 95% CI [0.01, 0.12], *p* = .011. Error bars represent 95 % confidence intervals.

We also created an additional dichotomous bluffing variable that was coded as “1” for participants who bluffed three or four times (“Heavy bluffers”) and “0” otherwise (“Light bluffers”). We then performed a two-step multiple logistic regression analysis predicting the dichotomous variable by (first step) entering a dummy-coded categorical “Avatar balance” variable (“All female”, “Gender mixed” and “All male” groups, with “All male” as the reference) and then (second step) Poker Experience Scale as independent variables into the model. Participants who did not recognize the avatar genders with 100% accuracy were removed from the analyses.

Of the participants, 24% were “Heavy bluffers”. In the first step of our analysis (Model 1), the average probabilities of being a “Heavy bluffer” when playing against all female avatar opponents (All female group) and all male opponents (All male group) were 0.299 and 0.202, respectively. In the second step (Model 2), after including Poker Experience Scale as a covariate, the above corresponding probabilities were 0.301 and 0.182. Thus, the odds of being a “Heavy bluffer” were higher when playing against all females than against all males (Model 1: OR = 1.68, 95% CI [0.98 – 2.88], *p* = .059. Model 2: OR = 1.99, 95% CI [0.14 – 3.5], *p* = .015). Table 2 summarizes the results.

Table 2. Two-step multiple logistic regression analysis predicting the dichotomous (bluffing) dependent variable.

| **Variable** | B | SE | | Wald | OR | 95% CI (OR) | *p* |
| --- | --- | --- | --- | --- | --- | --- | --- |
| *Model 1* |  | |  |  |  |  |  |
| Avatar Balance |  | |  |  |  |  |  |
| All female | 0.52 | 0.28 | | 3.58 | 1.68 | 0.98 – 2.88 | .059 |
| Gender mixed | 0.15 | 0.28 | | 0.31 | 1.17 | 0.68 – 2 | ns |
| *Model 2* |  | |  |  |  |  |  |
| Avatar Balance |  | |  |  |  |  |  |
| All female | 0.69 | 0.29 | | 5.87 | 1.99 | 1.14 – 3.5 | .015 |
| Gender mixed | 0.16 | 0.28 | | 0.31 | 1.17 | 0.67 – 2 | ns |
| Poker Experience Scale | 0.28 | 0.06 | | 22.8 | 1.32 | 1.2 – 1.48 | <.001 |

*Note.* Model 1 χ²(2) = 3.74, p = ns, BIC = 33.6 (Nagelkerke’s *R^2^* = .013). Model 2 χ²(3) = 28.5, *p* <.001, BIC = 240.5 (Nagelkerke’s *R^2^* = .094). For Avatar Balance, “All male” is the reference group.

**Results from Manipulation Checks**

Table 3. Mean attribute values (min = 1, max = 7) across the four opponent avatars were calculated separately for each experimental group (“all female”, “all male” and “gender mixed”).

|  | **All female** | | **All male** | | **Gender mixed** | |  |
| --- | --- | --- | --- | --- | --- | --- | --- |
|  | Mean | SD | Mean | SD | Mean | SD | **Post hoc (Bonferroni)** |
| Friendliness | 4 | 0.87 | 3.64 | 0.90 | 3.81 | 0.8 | Female > Male, p = .001 |
| Trustworthiness | 3.72 | 0.8 | 3.43 | 0.94 | 3.49 | 0.85 | Female > Male, p = .011 |
| Competence | 3.95 | 0.74 | 3.91 | 0.90 | 3.85 | 0.78 | ns |
| Warmth | 3.65 | 0.82 | 3.27 | 0.85 | 3.49 | 0.80 | Female > Male, p < .001 |
| Dominance | 3.58 | 0.79 | 3.53 | 0.9 | 3.53 | 0.86 | ns |
| Likability | 3.97 | 0.76 | 3.56 | 0.89 | 3.75 | 0.76 | Female > Male, p < .001 |
| Threat | 3.08 | 0.92 | 3.31 | 0.96 | 3.14 | 0.86 | ns |
| Attractiveness | 3.50 | 1 | 2.71 | 1.05 | 3.39 | 1.02 | Female > Male, p < .001 |

Mean attribute values (min = 1, max = 7) across the four opponent avatars were calculated separately for each experimental group (“all female”, “all male” and “gender mixed”). No significant differences in mean attributes were observed in “gender mixed vs. all male” or “gender mixed vs. all female” Bonferroni-corrected post hoc comparisons.

Pretests suggested that the avatars employed in the final experiments were on average similar with respect to all measured attributes. However, in the final sample, avatars in the “all female” group were reported to be more friendly, trustworthy, warm, likable and attractive than avatars in the “all male” group. Adding these measures as covariates in the experimental model did not cause any changes in the results.
